# Time-dependent changes in meibum lipid composition and progression of dry eye following disruption of the fatty acid elongase *Elovl1*

**DOI:** 10.1016/j.jbc.2026.111160

**Published:** 2026-01-13

**Authors:** Himari Tada, Keisuke Jojima, Taiga Hiranuma, Takayuki Sassa, Akio Kihara

**Affiliations:** Laboratory of Biochemistry, Faculty of Pharmaceutical Sciences, Hokkaido University, Sapporo, Japan

**Keywords:** dry eye, eye, fatty acid, gene KO, lipid, lipid metabolism, meibum, pathology, very-long-chain fatty acid, wax ester

## Abstract

The fatty acid (FA) elongase ELOVL1 is responsible for the synthesis of very-long-chain (VLC) FAs (VLCFAs) with carbon chain lengths of ≥C21. Meibum lipids, which constitute the lipid layer of the tear film, contain several lipid classes containing VLCFAs or their metabolites, VLC fatty alcohols. In this study, we investigated the short-, medium-, and long-term effects of *Elovl1* disruption on dry eye phenotypes and meibum lipid composition using two genetically engineered mouse models: epidermis-specific *Elovl1* transgene–containing *Elovl1* KO (*Tg-Elovl1* KO) mice and tamoxifen (Tam)-inducible *Elovl1* conditional KO mice. In *Tg-Elovl1* KO mice, shortening of the chain lengths of cholesteryl esters, wax monoesters, and type 1ω wax diesters, as well as a reduction in the quantity of type 2ω wax diesters, was observed. These changes were also observed in a time-dependent manner in *Elovl1* conditional KO mice administered Tam: in most cases, changes began on day 5 after the start of Tam administration, progressed rapidly until day 10, and then progressed more slowly thereafter. However, dry eye phenotypes (partially closed eyes, increased eye blink frequency, elevated water evaporation, plugging in the meibomian gland orifice, and tear film instability) were observed later than lipid composition changes, mostly starting from day 10 and becoming more pronounced by day 30. These findings reveal a link between alterations in meibum lipid composition and dry eye phenotypes and provide new insights into the metabolic flux of VLCFAs.

Of the fatty acids (FAs) that constitute lipids in living organisms, the majority are long-chain FAs with carbon chain lengths ranging from C11 to C20, with C16–C20 being particularly abundant (1, 2). Very-long-chain (VLC) fatty acids (VLCFAs; ≥C21) account for less than 10% of total FAs but are present in specific lipid classes in certain tissues and fulfill unique biological functions that cannot be substituted by long-chain FAs (3, 4). These VLCFA-containing lipid classes include ceramides (skin barrier formation), meibum lipids (protection of the cornea), polyunsaturated sphingolipids (spermatogenesis), polyunsaturated phosphatidylcholines (retinal and nerve functions), and saturated or monounsaturated sphingolipids (liver and myelin functions) (5, 6, 7, 8, 9, 10, 11, 12, 13, 14). The meibum lipids are a group of lipids that are secreted from the meibomian glands in the eyelids (15, 16).

VLCFAs are produced from long-chain FAs *via* the FA-elongation cycle in the endoplasmic reticulum (ER) (3, 4). This cycle consists of four reactions (condensation, reduction, dehydration, and reduction) and increases the carbon chain length of acyl-CoAs, the activated form of FAs, by two per cycle. The rate-limiting step in the FA-elongation cycle is the condensation reaction (17), which is catalyzed by FA elongases. Mammals have seven FA elongase isozymes (ELOVL1–ELOVL7), each of which shows substrate specificity toward acyl-CoAs with different chain lengths and degrees of unsaturation (18). For instance, ELOVL1 is active toward C18–C26 saturated and monounsaturated acyl-CoAs (production of C20–C28 acyl-CoAs), with particularly high activity toward C22–C24 acyl-CoAs (production of C24–C26 acyl-CoAs) (13, 18, 19). We previously generated whole-body KO mice of *Elovl1* and found that they exhibited neonatal lethality (death within 1 day of birth) because of skin barrier abnormalities caused by impaired production of VLCFA-containing ceramides (ω-*O*-acylceramides and protein-bound ceramides) (19, 20). We then generated *Elovl1* transgenic mice (*Tg*[*IVL-Elovl1*]) carrying an *Elovl1* transgene (*Tg*) under the control of an epidermis-specific involucrin (*IVL*) promoter and crossed them with heterozygous *Elovl1* KO (*Elovl1*^+/−^) mice to produce *Tg*(*IVL-Elovl1*) *Elovl1*^−/−^ mice (hereafter referred to as *Tg-Elovl1* KO mice) (13). In *Tg-Elovl1* KO mice, *Elovl1* is expressed only in the epidermis and is absent in other tissues. This prevented the skin barrier defects observed in whole-body *Elovl1* KO mice, enabling *Tg-Elovl1* KO mice to grow to adulthood. However, they exhibited dry eye and neurological abnormalities, which were caused by shortening of the chain lengths of meibum lipids in meibomian glands and sphingolipids (galactosylceramides, sulfatides, and sphingomyelins) in myelin, respectively (13, 14, 21). In humans, mutations in *ELOVL1* cause the neurocutaneous disease IKSHD syndrome (22, 23, 24). IKSHD is an acronym for the symptoms: *i*chthyotic *k*eratoderma, *s*pasticity, *h*ypomyelination, and *d*ysmorphia (22).

Tear film is composed of an outer lipid layer and an inner mucoaqueous layer (Fig. 1*A*) (25, 26). The lipid layer protects the cornea by preventing evaporation of water from the tears, reducing surface tension, providing appropriate viscoelasticity to the tears, and lubricating the gap between the eyelid and cornea (27, 28, 29, 30). There are two types of dry eye: evaporative dry eye and aqueous-deficient dry eye (31). Evaporative dry eye is caused by an abnormality in the lipid layer, often caused by some dysfunction in the meibomian glands, such as plugging in the meibomian gland orifices and atrophy of the meibomian glands (31, 32, 33, 34). Meibum lipids are major components of the tear film lipid layer and contain unique lipid classes, such as cholesteryl esters (Chol-Es), wax monoesters (WmEs), wax diesters (WdiEs), (*O*-acyl)-ω-hydroxy (ω-OH) FAs (OAHFAs), and cholesteryl OAHFAs (Chol-OAHFAs) (Fig. 1*B*) (15, 16, 35, 36, 37). These lipids contain VLCFAs or their derivatives, VLC fatty alcohols (FAls; VLCFAls). In a previous study, we measured Chol-Es and WmEs in the meibomian glands of *Tg-Elovl1* KO mice *via* LC–tandem MS (MS/MS) and found that the FA and FAl moieties, respectively, were shortened (13, 38).

**Figure 1:**
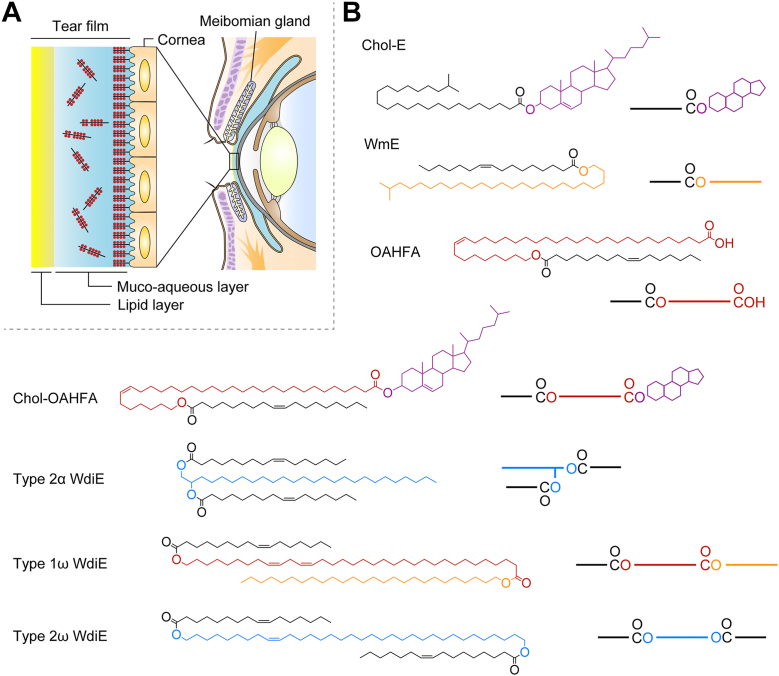
**Structures of tear film, eye, and meibum lipids**. *A*, schematic representation of the ocular surface, including the tear film, eye, and eyelids. Most of the lipids that constitute the lipid layer of the tear film are meibum lipids secreted from the meibomian glands located in the eyelids. *B*, structural formulae and schematic representation of meibum lipids. *Black*, FAs; *purple*, cholesterol; *orange*, FAls; *red*, ω-OH FAs; and *blue*, diols. Chol-E, cholesteryl ester; Chol-OAHFA, cholestery (*O*-acyl)-ω-hydroxy fatty acid; FA, fatty acid; FAl, fatty alcohol; OAHFA, (*O*-acyl)-ω-hydroxy fatty acid; WdiE, wax diester; WmE, wax monoester; ω-OH, ω-hydroxy.

In another attempt to develop a mouse strain that circumvents the neonatal lethality of *Elovl1* KO, we recently generated tamoxifen (Tam)-inducible *Elovl1* conditional KO (cKO; *Rosa26*^*+/Cre-ERT2*^ *Elovl1*^*flox/flox*^) mice using the *Cre*–*loxP* system, where *Cre-ERT2* is inserted into the *Rosa26* locus (39). Since *Tg-Elovl1* KO mice lack *Elovl1* from the developmental stage, the maximum effect of *Elovl1* KO should be observable. However, we cannot exclude the possibility that some of the dry eye phenotypes observed may have been affected by compensatory responses, such as the expression of other genes. Indeed, increased expression of *Elovl3*, *Elovl4*, and *Elovl7*, which function redundantly with *Elovl1* in the production of VLCFAs, has been observed in *Tg-Elovl1* KO mice (13). However, *Elovl1* cKO mice become deficient in *Elovl1* only after Tam administration, allowing us to examine the early effects (with fewer compensatory effects) of *Elovl1* KO on the already-formed tear film lipid layer. Furthermore, we can also observe the time-dependent effects of *Elovl1* KO using these mice. In this study, we used both mouse strains to investigate the effects of long-term *Elovl1* deficiency (*Tg-Elovl1* KO mice) and short- to medium-term and time-dependent *Elovl1* deficiency (*Elovl1* cKO mice). Through these analyses, we obtained insights into the relationship between changes in meibum lipid composition caused by impaired VLCFA and VLCFAl production and various dry eye phenotypes.

## Results

### Shortening of the FAl moiety of type 1ω WdiEs and reduced quantity of type 2ω WdiEs in *Tg-Elovl1* KO mice

We previously reported that the FA moiety of Chol-Es and the FAl moiety of WmEs were shortened in *Tg-Elovl1* KO mice relative to control mice (*Tg-Elovl1*^+/+^), whereas the composition of OAHFAs remained almost unchanged (13). In that report, WdiEs and Chol-OAHFAs were not measured because the measurement method had not yet been established. However, we subsequently established an LC–MS/MS system in multiple reaction monitoring (MRM) mode that could measure these lipids and revealed the presence and detailed composition of WdiEs (type 1ω, type 2α, and type 2ω) and Chol-OAHFAs in mouse meibum lipids (37, 40). In this study, we measured these lipids to elucidate the whole picture of meibum lipid composition in *Tg-Elovl1* KO mice using this newly established system.

**Figure 2:**
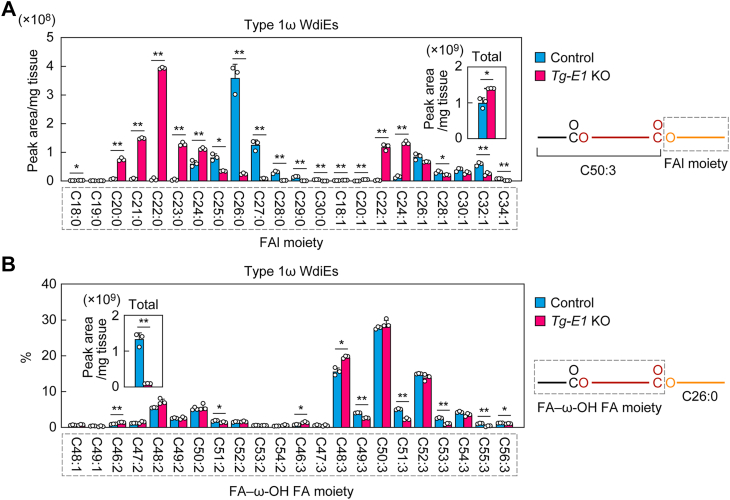
**Shortening of the FAl moiety of type 1ω WdiEs in *Tg-Elovl1* KO mice**. Lipids were extracted from the eyelids of 8-week-old control mice (n = 3; 1 male, 2 females) and *Tg-Elovl1* KO mice (n = 3; all females), and type 1ω WdiEs containing a C50:3 FA–ω-OH FA moiety (*A*) or C26:0 FAl moiety (*B*) were measured *via* LC–MS/MS. The quantity of each FAl species (*A*), the percentage of each FA-ω-OH FA species (*B*), and their total quantities (*A* and *B*; *insets*) are shown. Values presented are mean + SD (∗*p* < 0.05, *∗∗p* < 0.01; Welch’s *t* test). The simplified structures of the type 1ω WdiEs, showing the analyzed moieties, are presented. *E1*, *Elovl1*; FA, fatty acid; FAl, fatty alcohol; MS/MS, tandem MS; *Tg*, transgene; WdiE, wax diester; ω-OH, ω-hydroxy.

We first extracted lipids from the eyelids (which contained meibomian glands) of control mice and *Tg-Elovl1* KO mice and measured type 1ω WdiEs *via* LC–MS/MS in MRM mode using a triple quadrupole (Q) mass spectrometer. Type 1ω WdiEs are composed of an FA, an ω-OH FA, and an FAl, with the ω-OH FA being esterified to the FAl and FA at the 1- and ω-positions, respectively. In MRM analysis, where the *m/z* values of the precursor ion to be selected in Q1 and the product ion to be selected in Q3 are specified, the target molecule can be measured by dividing it into two characteristic components. For example, in the case of type 1ω WdiEs, by setting the *m/z* of the precursor ion [M + H]^+^ in Q1 and that of the product ion [M + H − FAl]^+^ in Q3, type 1ω WdiE species with a specific FAl and a specific FA–ω-OH FA moiety can be measured (37). We previously reported that the most abundant FA–ω-OH FA moiety in type 1ω WdiEs in mouse meibum lipids was C50:3 (37). Based on this information, we now examined the FAl composition of type 1ω WdiE containing the C50:3 FA–ω-OH FA moiety in *Tg-Elovl1* KO mice. We found that the FAl moiety was shortened in *Tg-Elovl1* KO mice relative to control mice (Fig. 2*A*). The extent of the shortening was greater in saturated FAls than in monounsaturated FAls. The most abundant FAl moiety in control mice was C26:0, followed by C27:0. In *Tg-Elovl1* KO mice, the quantities of type 1ω WdiE containing ≥C25:0 FAls were reduced, whereas those with ≤C24:0 FAls were increased. The total quantity of type 1ω WdiEs containing the C50:3 FA–ω-OH FA moiety was slightly increased in *Tg-Elovl1* KO mice relative to control mice (Fig. 2*A*, *inset*).

We next examined the FA–ω-OH FA composition of type 1ω WdiEs containing C26:0 FAl, which was the most abundant FAl moiety in control mice (Fig. 2*A*). In *Tg-Elovl1* KO mice, the total quantity of type 1ω WdiEs containing C26:0 FAl was reduced to 7.0% of that in control mice (Fig. 2*B*, *inset*), which was consistent with the aforementioned finding that the type 1ω WdiE species consisting of C50:3 FA-ω-OH FA and C26 FAl was similarly reduced in *Tg-Elovl1* KO mice (7.1% of levels in control mice). In contrast, the composition of FA–ω-OH FA species (the ratio of each FA–ω-OH FA species to the total quantity of type 1ω WdiEs containing C26:0 FAl) was similar in control and *Tg-Elovl1* KO mice (Fig. 2*B*). These results indicate that the FAl moiety but not the FA–ω-OH FA moiety was shortened in type 1ω WdiEs in *Tg-Elovl1* KO mice.

**Figure 3:**
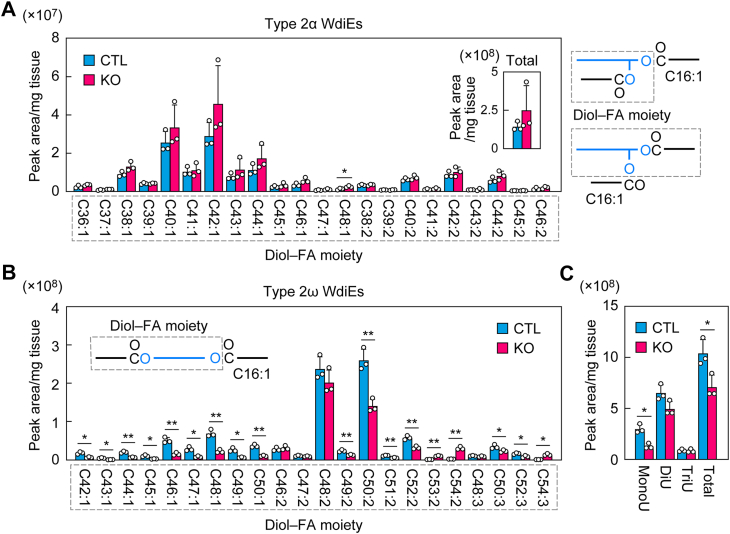
**Decrease in the quantity of monounsaturated diol–FA-containing type 2 ω WdiEs in *Tg-Elovl1* KO mice**. Lipids were extracted from the eyelids of 8-week-old control mice (n = 3; 1 male, 2 females) and *Tg-Elovl1* KO mice (n = 3; all females), and C16:1 FA-containing type 2α (*A*) and type 2ω (*B* and *C*) WdiEs were measured *via* LC–MS/MS. The quantity of each diol–FA species of type 2α WdiEs (*A*) or type 2ω WdiEs (*B*), total quantity of type 2α WdiEs (*A*, *inset*), total quantities of type 2ω WdiEs containing mono-, di-, and triunsaturated diol–FA moieties (*C*), and total quantity of type 2ω WdiEs (*C*) are shown. Values presented are mean + SD (∗*p* < 0.05, *∗∗p* < 0.01; Welch’s *t* test). The simplified structures of the type 2α and type 2ω WdiEs, showing the analyzed moiety, are presented (*A* and *B*). CTL, control; DiU, diunsaturated; FA, fatty acid; MonoU, monounsaturated; MS/MS, tandem MS; *Tg*, transgene; TriU, triunsaturated; WdiE, wax diester.

Type 2 WdiEs consist of one diol (type 2α WdiEs, 1,α-diol; type 2ω WdiEs, 1,ω-diol) and two FAs, each of which is ester linked to one of the hydroxyl groups of the diol. In mouse meibum lipids, the most predominant FA moiety is C16:1 in both type 2α WdiEs and type 2ω WdiEs (37, 40). Therefore, we investigated the diol–FA composition of type 2 WdiEs containing C16:1 FA. LC–MS/MS in MRM mode revealed that both the total quantity and the diol–FA composition of type 2α WdiEs containing C16:1 FA were similar in control and *Tg-Elovl1* KO mice (Fig. 3*A*). In both strains, the most abundant diol–FA moiety was C42:1, followed by C40:1.

In contrast, many of the type 2ω WdiE species were reduced in *Tg-Elovl1* KO mice relative to control mice (Fig. 3*B*). Type 2ω WdiEs contained mono-, di-, and triunsaturated diol–FA moieties. Of these, the reduction was most pronounced in type 2ω WdiEs containing monounsaturated diol–FAs. The total quantity of type 2ω WdiEs containing monounsaturated diol–FAs in *Tg-Elovl1* KO mice was 43% of that in control mice, whereas quantities of type 2ω WdiEs containing di- or triunsaturated diol–FAs were comparable between control and *Tg-Elovl1* KO mice (Fig. 3*C*). The total quantity of type 2ω WdiEs (the sum of the quantities of all the above type 2ω WdiEs containing mono-, di-, and triunsaturated diol–FAs) in *Tg-Elovl1* KO mice was 68% of that in control mice.

**Figure 4:**
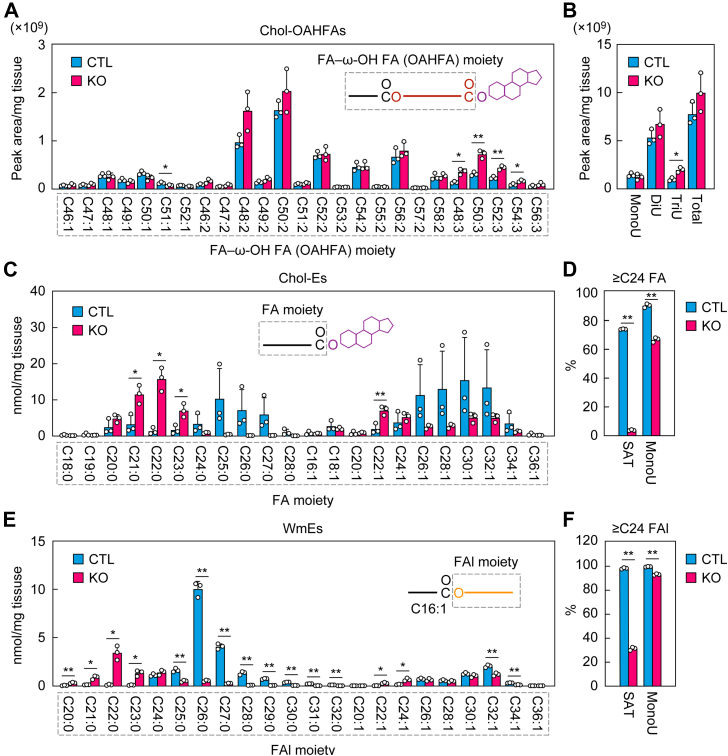
**Shortening of the FA moiety of Chol-Es and the FAl moiety of WmEs in *Tg-Elovl1* KO mice**. Lipids were extracted from the eyelids of 8-week-old control mice (n = 3; 1 male, 2 females) and *Tg-Elovl1* KO mice (n = 3; all females), and Chol-OAHFAs (*A* and *B*), Chol-Es (*C* and *D*), and C16:1 FA-containing WmEs (*E* and *F*) were measured *via* LC–MS/MS. The quantity of each OAHFA, FA, or FAl species (*A*, *C*, and *E*), total quantities of Chol-OAHFAs containing mono-, di-, and triunsaturated OAHFA moieties (*B*), total quantity of Chol-OAHFAs (*B*), and percentages of Chol-Es (*D*) or WmEs (*F*) containing saturated or monounsaturated ≥C24 FA or FAl moieties, respectively, are shown. Values presented are mean + SD (∗*p* < 0.05, *∗∗p* < 0.01; Welch’s *t* test). The simplified structures of Chol-OAHFA, Chol-E, and WmE, showing the analyzed moiety, are presented (*A*, *C*, and *E*). Chol-E, cholesteryl ester; Chol-OAHFA, cholesteryl (*O*-acyl)-ω-hydroxy fatty acid; CTL, control; DiU, diunsaturated; FA, fatty acid; FAl, fatty alcohol; MonoU, monounsaturated; MS/MS, tandem MS; OAHFA, (*O*-acyl)-ω-hydroxy fatty acid; SAT, saturated; *Tg*, transgene; TriU, triunsaturated; WmE, wax monoester; ω-OH, ω-hydroxy.

Chol-OAHFAs are composed of cholesterol and an OAHFA (FA–ω-OH FA ester). We next investigated the OAHFA composition of Chol-OAHFAs in the meibum lipids of control and *Tg-Elovl1* KO mice *via* LC–MS/MS. Chol-OAHFAs contained mono-, di-, and triunsaturated OAHFAs, and the quantities of Chol-OAHFAs containing mono- and diunsaturated OAHFAs were comparable between control mice and *Tg-Elovl1* KO mice (Fig. 4, *A* and *B*). In contrast, the quantities of Chol-OAHFAs containing triunsaturated OAHFAs were increased in many species, and the total quantity in *Tg-Elovl1* KO mice was approximately double that in control mice. This increase may have been caused indirectly by changes in the quantities or composition of other meibum lipids. The total quantity of Chol-OAHFAs in *Tg-Elovl1* KO mice was not statistically different from that in control mice (Fig. 4*B*).

We have already reported the quantities and compositions of Chol-Es and WmEs in *Tg-Elovl1* KO mice (13, 38). However, in this study, we remeasured them, because the measurement conditions had been improved since that study was conducted (through, *e*.*g*., inclusion of a purification step before LC–MS/MS and optimization of MS/MS conditions). Chol-Es consist of cholesterol and a saturated or monounsaturated FA. In control mice, the predominant FAs in Chol-E were C25:0–C27:0 for saturated FAs and even-numbered C26:1–C32:1 for monounsaturated FAs (Fig. 4*C*). The FA moiety in *Tg-Elovl1* KO mice was shortened compared with that in control mice, and this shortening was more pronounced in saturated than in monounsaturated FA. The percentage of Chol-Es containing ≥C24 FAs was 74.1% for saturated FAs and 90.5% for monounsaturated FAs in control mice, whereas in *Tg-Elovl1* KO mice, it was 3.9% and 66.8%, respectively (Fig. 4*D*).

WmEs consist of an FA and an FAl. Since the most abundant FA moiety of WmEs in mouse meibum lipids is C16:1, followed by C18:1 (16, 37), we measured C16:1 FA-containing WmEs in this study. The FAl moiety in control mice was either saturated or monounsaturated, and WmEs containing saturated FAls were more abundant than those containing monounsaturated FAls (Fig. 4*E*). The most abundant FAl moieties were C26:0 for saturated FAls and C32:1 for monounsaturated FAls, followed by C27:0 and C30:1, respectively. The FAl portion was shortened in *Tg-Elovl1* KO mice relative to control mice, especially in the saturated FAl moiety. In control mice, the percentage of WmEs containing ≥C24 FAls was 98.3% for saturated FAls and 99.7% for monounsaturated FAls, whereas in *Tg-Elovl1* KO mice, it was 32.6% and 93.2%, respectively (Fig. 4*F*). These findings on the shortening of Chol-Es and WmEs in *Tg-Elovl1* KO mice were consistent with previous reports (13, 38). In summary, shortening of the FA or FAl moiety of Chol-Es, WmEs, and type 1ω WdiEs and reduced quantities of type 2ω WdiEs were observed in the meibum lipids of *Tg-Elovl1* KO mice.

### Time course of dry eye phenotypes in Tam-induced *Elovl1* cKO mice

**Figure 5:**
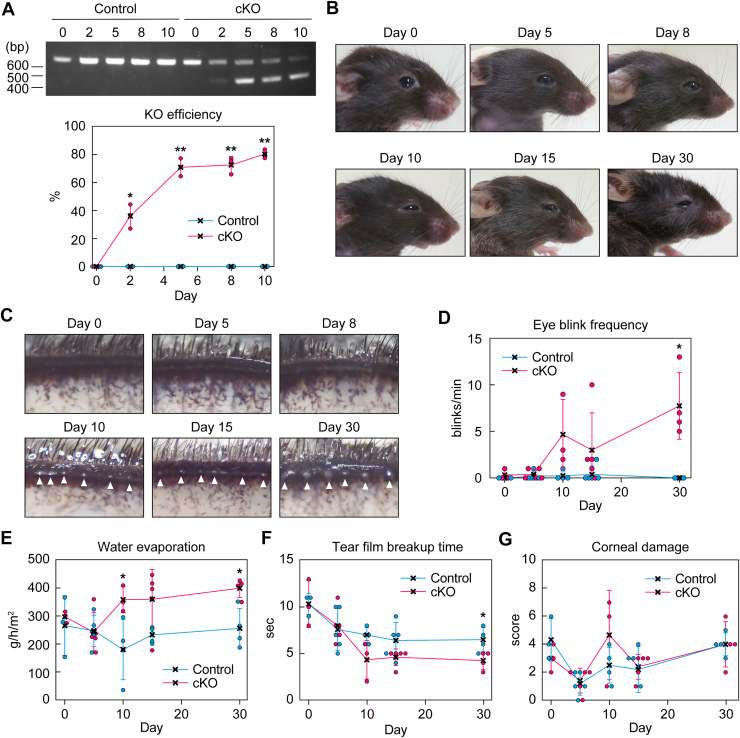
**Time-dependent development of dry eye phenotypes in *Elovl1* cKO mice**. *A*–*G*, control and *Elovl1* cKO mice at 3 months of age were intraperitoneally administered Tam (2 mg per dose) once daily for 5 consecutive days. *A*, genomic DNA was prepared from the eyelids of the mice (n = 3) on day 0 (control and cKO 2 males, 1 female each), 2 (control and cKO 3 males each), 5 (control and cKO 2 males, 1 female each), 8 (control and cKO 3 males each), and 10 (control 2 males, 1 female and cKO 3 females) after the initiation of Tam administration and subjected to genomic PCR. PCR fragments were separated *via* electrophoresis using agarose gel containing ethidium bromide, detected by exposing them to ultraviolet light, photographed, and quantified. Values presented are mean ± SD (∗*p* < 0.05, ∗∗*p* < 0.01; Welch’s *t* test). *B* and *C*, photographs of faces (*B*) and upper eyelids (*C*) of *Elovl1* cKO mice on day 0, 5, 8, 10, 15, and 30 after the initiation of Tam administration are shown. *Arrowheads* represent plugging of the meibomian gland orifices (*C*). *D–G*, the eye blink frequency (*D*), water evaporation from the ocular surface (*E*), BUT (*F*), and corneal damage score (*G*) were measured in mice (n = 3–5) on day 0 (control 3 males and cKO 2 males, 1 female), 5 (control 1 male, 4 females and cKO 5 females), 10 (control 2 males, 2 females and cKO 2 males, 1 female), 15 (control 4 males, 1 female and cKO 5 males), and 30 (control 3 males, 1 female and cKO 1 male, 3 females) after the initiation of Tam administration. Values presented are mean ± SD (∗*p* < 0.05; Welch’s *t* test). BUT, breakup time; cKO, conditional KO; Tam, tamoxifen.

To induce *Elovl1* deletion, 3-month-old *Elovl1* cKO mice were intraperitoneally administered 2 mg of Tam once daily for 5 consecutive days. We first examined the KO efficiency of *Elovl1* in the eyelids *via* genomic PCR. The KO efficiency increased to approximately 40% on day 2 and reached approximately 70% on day 5 from the start of Tam administration, after which it remained almost constant (Fig. 5*A*). This result indicates that the KO of *Elovl1* in the eyelids was almost complete within 5 days of the start of Tam administration.

We previously reported that *Tg-Elovl1* KO mice exhibited various dry eye phenotypes, such as partially closed eyes, increased blink rate, elevated water evaporation, plugging in the meibomian gland orifice, increased tear film instability (shortened tear film breakup time [BUT]), and corneal damage (13, 21). In this study, to reveal the time course of dry eye progression, we examined these phenotypes in *Elovl1* cKO mice from day 0 to day 30 after the start of Tam administration. From day 10 onward, the eyes of *Elovl1* cKO mice began to partially close and tears accumulated on the lower eyelids (Fig. 5*B*), the latter suggesting that the surface tension of the tears was elevated. Plugging in the meibomian gland orifices was also observed from day 10 onward (Fig. 5*C*). Mice normally blink rarely, and the number of blinks per minute in control mice was almost zero throughout the observation period (Fig. 5*D*). In contrast, almost all the *Elovl1* cKO mice blinked multiple times per minute from day 10 onward, and the average number of blinks on day 30 was approximately eight. Water evaporation from the eyes was increased in *Elovl1* cKO mice from day 10 onward relative to control mice (Fig. 5*E*). The BUT of *Elovl1* cKO mice tended to be shorter than that of control mice from day 10 onward, but this difference was only statistically significant on day 30 (Fig. 5*F*). However, corneal damage scores were similar between control and *Elovl1* cKO mice even on day 30 (Fig. 5*G*). In summary, most of the dry eye phenotypes in *Elovl1* cKO mice were observed on day 10 from the start of Tam administration, but it may take longer than 30 days for corneal damage to occur.

### Time course of compositional changes in meibum lipids in *Elovl1* cKO mice

**Figure 6:**
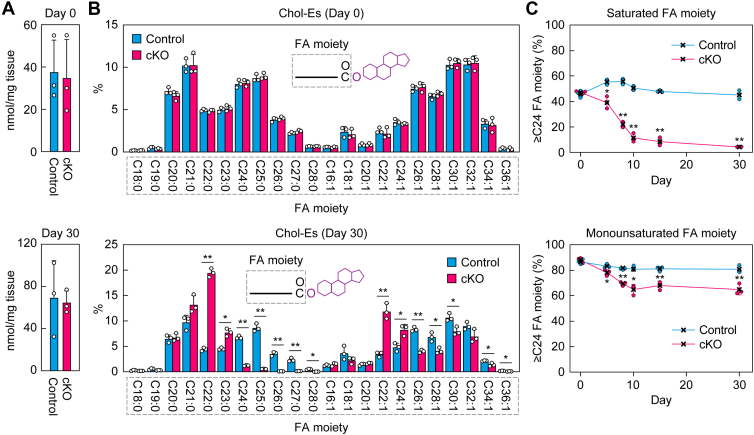
**Time-dependent shortening of the FA moiety of Chol-Es in *Elovl1* cKO mice**. Control mice (n = 3) and *Elovl1* cKO mice (n = 3) at 3 months of age were intraperitoneally administered Tam (2 mg per dose) once daily for 5 consecutive days. Lipids were extracted from the eyelids of mice on days 0 (control and cKO 3 males each), 5 (control 1 male, 2 females and cKO 3 females), 8 (control 1 male, 2 females and cKO 2 males, 1 female), 10 (control 1 male, 2 females and cKO 2 males, 1 female), 15 (control and cKO 3 males each), and 30 (control 2 males, 1 female and cKO 3 females) after the initiation of Tam administration, and Chol-Es were measured *via* LC–MS/MS. The total quantity of Chol-Es (*A*) and the quantity of each FA species (*B*) on days 0 (*upper graphs*) and 30 (*lower graphs*), and percentages of Chol-Es containing saturated *(upper graph*) or monounsaturated (*lower graph*) FA moieties with ≥C24 chain lengths on days 0, 5, 8, 10, 15, and 30 (*C*) are shown. Values presented are mean + SD (*A* and *B*) or ± SD (*C*) (∗*p* < 0.05, *∗∗p* < 0.01; Welch’s *t* test). The simplified structures of Chol-Es, showing the analyzed moieties, are presented (*B*). Chol-E, cholesteryl ester; cKO, conditional KO; FA, fatty acid; MS/MS, tandem MS; Tam, tamoxifen.

**Figure 7:**
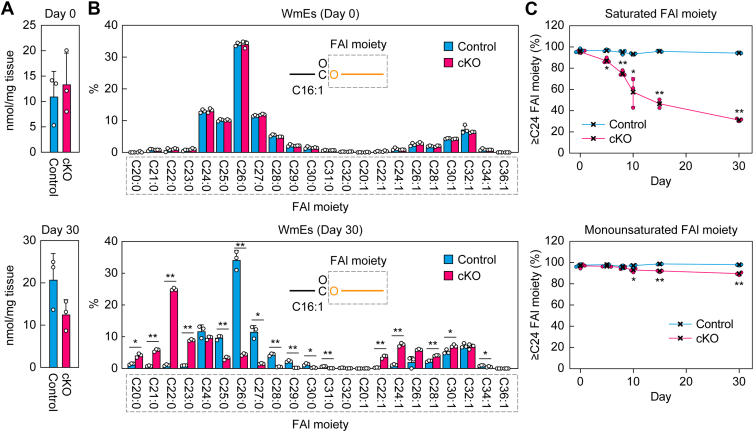
**Time-dependent shortening of the FAl moiety of WmEs in *Elovl1* cKO mice**. Control mice (n = 3) and *Elovl1* cKO mice (n = 3) at 3 months of age were intraperitoneally administered Tam (2 mg per dose) once daily for 5 consecutive days. Lipids were extracted from the eyelids of mice on days 0 (control and cKO 3 males each), 5 (control 1 male, 2 females and cKO 3 females), 8 (control 1 male, 2 females and cKO 2 males, 1 female), 10 (control 1 male, 2 females and cKO 2 males, 1 female), 15 (control and cKO 3 males each), and 30 (control 2 males, 1 female and cKO 3 females) after the initiation of Tam administration, and C16:1 FA-containing WmEs were measured *via* LC–MS/MS. The total quantity of WmEs (*A*) and the quantity of each FAl species (*B*) on days 0 (*upper graphs*) and 30 (*lower graphs*), and the percentages of WmEs containing saturated (*upper graph*) or monounsaturated (*lower graph*) FAl moieties with ≥C24 chain lengths on days 0, 5, 8, 10, 15, and 30 (*C*) are shown. Values presented are mean + SD (*A* and *B*) or ± SD (*C*) (∗*p* < 0.05, *∗∗p* < 0.01; Welch’s *t* test). The simplified structures of WmEs, showing the analyzed moieties, are presented in (*B*). cKO, conditional KO; FAl, fatty alcohol; MS/MS, tandem MS; Tam, tamoxifen; WmE, wax monoester.

**Figure 8:**
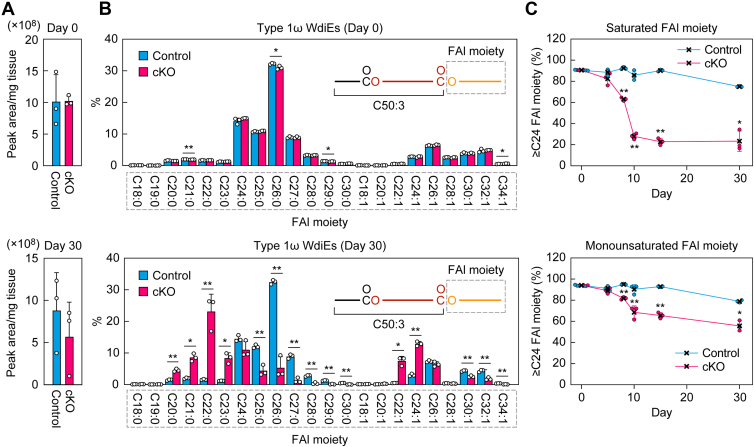
**Time-dependent shortening of the FAl moiety of type 1ω WdiEs in *Elovl1* cKO mice**. Control mice (n = 3) and *Elovl1* cKO mice (n = 3) at 3 months of age were intraperitoneally administered Tam (2 mg per dose) once daily for 5 consecutive days. Lipids were extracted from the eyelids of mice on days 0 (control and cKO 3 males each), 5 (control 1 male, 2 females and cKO 3 females), 8 (control 1 male, 2 females and cKO 2 males, 1 female), 10 (control 1 male, 2 females and cKO 2 males, 1 female), 15 (control and cKO 3 males each), and 30 (control 2 males, 1 female and cKO 3 females) after the initiation of Tam administration, and C50:3 FA–ω-OH FA-containing type 1ω WdiEs were measured *via* LC–MS/MS. The total quantity of type 1ω WdiEs (*A*) and the quantity of each FAl species (*B*) on days 0 (*upper graphs*) and 30 (*lower graphs*), and the percentages of type 1ω WdiEs containing saturated (*upper graph*) or monounsaturated (*lower graph*) FAl moieties with ≥C24 chain lengths on days 0, 5, 8, 10, 15, and 30 (*C*) are shown. Values presented are mean + SD (*A* and *B*) or ±SD (*C*) (∗*p* < 0.05, *∗∗p* < 0.01; Welch’s *t* test). The simplified structures of type 1ω WdiEs, showing the analyzed moieties, are presented in (*B*). cKO, conditional KO; FA, fatty acid; FAl, fatty alcohol; MS/MS, tandem MS; Tam, tamoxifen. WdiE, wax diester; ω-OH, ω-hydroxy.

**Figure 9:**
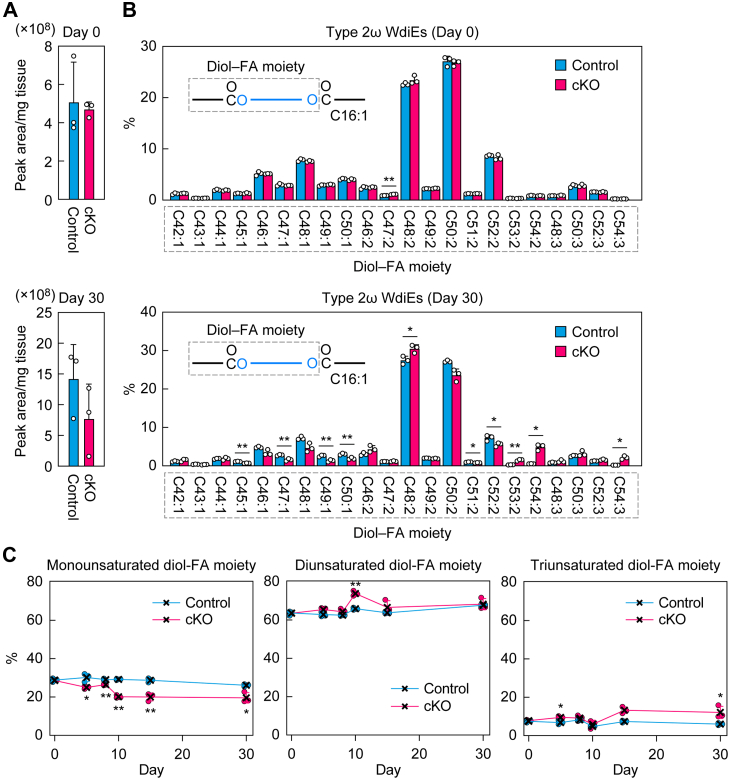
**Time-dependent decrease in the quantity of monounsaturated diol–FA-containing type 2ω WdiEs in *Elovl1* cKO mice**. Control mice (n = 3) and *Elovl1* cKO mice (n = 3) at 3 months of age were intraperitoneally administered Tam (2 mg per dose) once daily for 5 consecutive days. Lipids were extracted from the eyelids of mice on days 0 (control and cKO 3 males each), 5 (control 1 male, 2 females and cKO 3 females), 8 (control 1 male, 2 females and cKO 2 males, 1 female), 10 (control 1 male, 2 females and cKO 2 males, 1 female), 15 (control and cKO 3 males each), and 30 (control 2 males, 1 female and cKO 3 females) after the initiation of Tam administration, and C16:1 FA-containing type 2ω WdiEs were measured *via* LC–MS/MS. The total quantity of type 2ω WdiEs (*A*) and the quantity of each diol–FA species (*B*) on days 0 (*upper graphs*) and 30 (*lower graphs*), and the percentages of type 2ω WdiEs containing mono-, di-, and triunsaturated diol–FA moieties on days 0, 5, 8, 10, 15, and 30 (*C*) are shown. Values presented are mean + SD (*A* and *B*) or ± SD (*C*) (∗*p* < 0.05, *∗∗p* < 0.01; Welch’s *t* test). The simplified structures of type 2ω WdiEs, showing the analyzed moieties, are presented in (*B*). cKO, conditional KO; FA, fatty acid; MS/MS, tandem MS; WdiE, wax diester; Tam, tamoxifen.

To reveal the relationship between the progression of dry eye and changes in meibum lipid composition in *Elovl1* cKO mice, the meibum lipids affected in *Tg-Elovl1* KO mice (Chol-Es, WmEs, type 1ω WdiEs, and type 2ω WdiEs; Figure 2, Figure 3, Figure 4) were measured *via* LC–MS/MS in MRM mode. On day 0 after Tam administration, the total quantities of Chol-Es (Fig. 6*A*), WmEs containing a C16:1 FA moiety (Fig. 7*A*), type 1ω WdiEs containing a C50:3 FA–ω-OH FA moiety (Fig. 8*A*), and type 2ω WdiEs containing a C16:1 FA moiety (Fig. 9*A*), as well as their FA (Fig. 6*B*), FAl (Figs. 7*B* and 8*B*), or diol–FA (Fig. 9*B*) composition, were all comparable between control and *Elovl1* cKO mice. The total quantities remained similar in these strains on day 30 after Tam administration (Figs. 6*A*–9*A*). In contrast, the composition of these meibum lipids changed in *Elovl1* cKO mice in a time-dependent manner following Tam treatment. For Chol-Es, shortening of the FA moieties was observed from day 5, progressed until day 10, and then reached a plateau in *Elovl1* cKO mice (Fig. 6*C*). This shortening was more pronounced in saturated FA than in monounsaturated FA moieties (Fig. 6, *B* and *C*). The percentage of Chol-Es containing ≥C24 FAs was 47% on day 0 and decreased to 4% on day 30 for saturated FA–containing Chol-Es, whereas it was 88% on day 0 and 65% on day 30 for monounsaturated FA–containing Chol-Es (Fig. 6*C*).

The shortening of the FAl moieties of WmEs in *Elovl1* cKO mice was also more marked in saturated than in monounsaturated FAl moieties (Fig. 7, *B* and *C*). The shortening of saturated FAl moieties was observed from day 5 and continued through day 30 (Fig. 7*C*). The percentage of WmEs containing saturated ≥C24 FAl moieties was 96% on day 0 and 31% on day 30. In contrast, the shortening of monounsaturated FAl moieties began from day 10 and progressed weakly through day 30. The percentage of WmEs containing monounsaturated ≥C24 FAl moieties was 97% on day 0 and 90% on day 30.

Similarly, the shortening of the FAl moieties of the C50:3 FA–ω-OH FA-containing type 1ω WdiEs in *Elovl1* cKO mice was more pronounced in saturated than in monounsaturated FAl moieties (Fig. 8, *B* and *C*). Both shortening processes began on day 8, progressed through day 10, and remained almost unchanged thereafter. The percentage of saturated ≥C24 FAl moieties was 91% on day 0 and 23% on day 30 (Fig. 8*C*). In contrast, that of monounsaturated ≥C24 FAl moieties was 94% on day 0 and 56% on day 30. To summarize these results, in Chol-Es, WmEs, and type 1ω WdiEs, saturated FA/FAl moieties were more susceptible to chain shortening by *Elovl1* KO than monounsaturated FA/FAl moieties.

In *Tg-Elovl1* KO mice, the total quantity of type 2ω WdiEs containing monounsaturated diol–FA moieties was reduced (43% of control mice) (Fig. 3, *B* and *C*). In *Elovl1* cKO mice, that quantity decreased gradually from day 5 after Tam administration, reaching 75% of that in control mice on day 30 (Fig. 9, *B* and *C*). In contrast, the total quantities of type 2ω WdiEs containing di- and triunsaturated diol–FA moieties were unaffected or only slightly affected by *Elovl1* KO throughout the analytical period (Fig. 9*C*).

### Gene expression changes in *Elovl1* cKO mice

**Figure 10:**
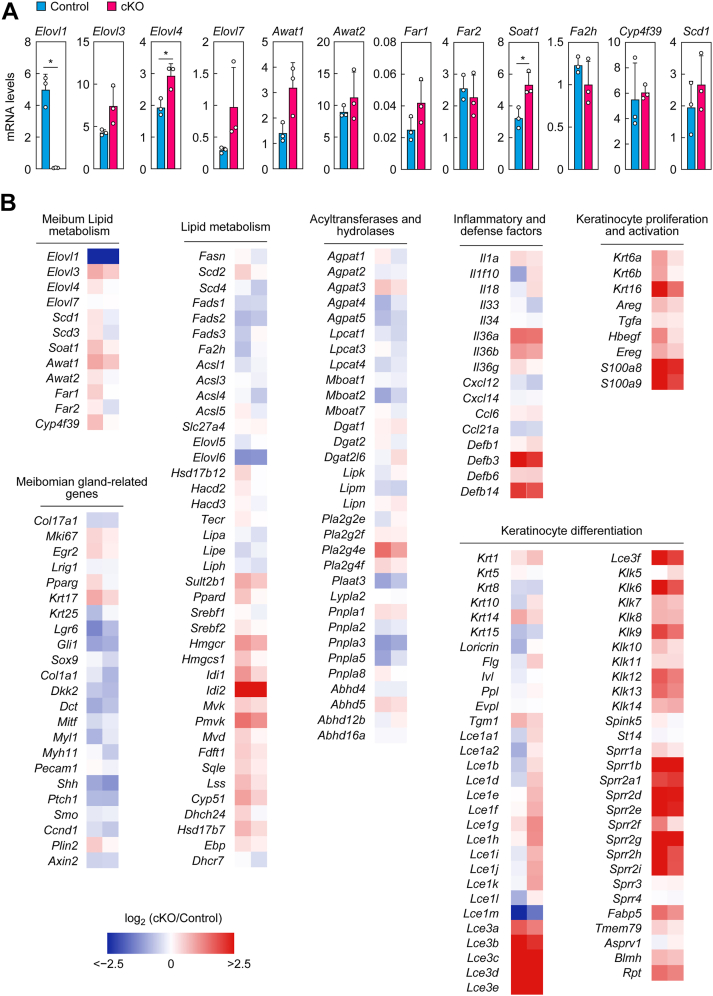
**Changes in expression of the genesin the eyelids of *Elovl1* cKO mice**. Control mice (*A*, n = 3 [1 male, 2 females]; *B*, n = 2 [2 females]) and *Elovl1* cKO mice (*A*, n = 3 [3 females]; *B*, n = 2 [2 females]) at 3 months of age were intraperitoneally administered Tam (2 mg per dose) once daily for 5 consecutive days. Total RNAs were prepared from the eyelids on day 30 after the initiation of Tam administration. *A*, the expression levels of the genes involved in meibum lipid syntheses and the housekeeping gene *Hprt1* were measured *via* quantitative real-time RT–PCR. Values presented are mean + SD of the mRNA levels relative to *Hprt1* (∗*p* < 0.05; Welch’s *t* test). *B*, total RNAs were subjected to RNA sequencing. The ratios of transcripts per million (TPMs) for each of the two *Elovl1* cKO mice to the mean TPM for the control mice are shown as a heatmap. Genes associated with meibum lipid metabolism, meibomian gland–related processes, lipid metabolism, acyltransferases and hydrolases, inflammatory and defense factors, keratinocyte proliferation and activation, and keratinocyte differentiation that have a TPM ≥10 are shown. cKO, conditional KO; Tam, tamoxifen.

We previously examined the expression levels of *Elovls* in the eyelids of control and *Tg*-*Elovl1* KO mice (13). Of *Elovls*, the expression levels of *Elovl1* were the highest in control mice, followed by *Elovl3* and *Elovl4*. Other *Elovls* were expressed only at low levels or were undetectable. In *Tg-Elovl1* KO mice, the expression levels of *Elovl3* and *Elovl7* were increased relative to control mice as a compensatory mechanism for *Elovl1* deletion. In this study, to investigate the compensatory responses in *Elovl1* cKO mice, we prepared RNAs from the eyelids of control and *Elovl1* cKO mice on day 30 after the start of Tam administration and measured the mRNA levels of the genes involved in meibum lipid biogenesis *via* quantitative real-time RT–PCR. The genes examined were the FA elongases *Elovl1*, *Elovl3*, *Elovl4*, and *Elovl7*, sterol *O*-acyltransferase *Soat1* (involved in Chol-E production) (41), acyl-CoA wax alcohol acyltransferases *Awat1* and *Awat2* (involved in ester bond formation in WmEs and WdiEs) (37), fatty acyl-CoA reductases *Far1* and *Far2* (involved in FAl production) (42), cytochrome P450 member *Cyp4f39* (responsible for ω-hydroxylation of the ω-OH FA-containing meibum lipids OAHFAs, Chol-OAHFAs, and type 1ω/2ω WdiEs) (40), FA 2-hydroxylase *Fa2h* (possibly involved in 2-hydroxylation of the FA in type 2α WdiEs) (43), and stearoyl-CoA desaturase *Scd1* (involved in the production of monounsaturated FA) (44). The expression levels of *Elovl1* in the meibomian glands of *Elovl1* cKO mice were reduced to 1.2% of those of control mice (Fig. 10*A*). The expression levels of *Elovl4* and *Soat1* were increased 1.5- and 1.6-fold, respectively, in *Elovl1* cKO mice compared with control mice. Those of *Elovl3*, *Elovl7*, *Awat1*, and *Far1* in *Elovl1* cKO mice also increased, but these differences were not statistically significant. The expression levels of the other genes were similar in control and *Elovl1* cKO mice. The increases in the expression levels of *Elovl3* and *Elovl7* in *Elovl1* cKO mice were smaller than in *Tg-Elovl1* KO mice, which showed 3.0- and 2.8-fold increases, respectively, compared with control mice (13). This result suggests that the compensatory responses had already begun on day 30 after the start of Tam treatment in *Elovl1* cKO but were ongoing.

Next, to comprehensively assess gene expression changes in *Elovl1* cKO mice, we performed RNA sequencing. Overall, changes in eyelid gene expression were less pronounced than those previously observed in the skin (39), both in terms of the number of affected genes and the magnitude of their changes. In the eyelids of *Elovl1* cKO mice, genes with expression changes greater than fivefold or less than 20% of control levels were *Idi2*, *Defb3*, *Defb14*, *Krt16*, *S100a8*, *S100a9*, and members of the *Lce3* (*Lce3b–e*) and *Sprr* families (*Sprr1b*, *Sprr2a1*, *Sprr2d*, *e*, *g*, *h*, and *i*) (Fig. 10*B*). No acyltransferases/hydrolases or meibomian gland–related genes exhibited changes beyond these thresholds. *Idi2* encodes isopentenyl-diphosphate isomerase, which catalyzes the isomerization of isopentenyl diphosphate to dimethylallyl diphosphate in isoprenoid and cholesterol biosyntheses (45). *Idi2* upregulation may represent a compensatory response to Chol-E chain shortening in meibocytes; however, Chol-E synthesis was not increased at least on day 30 after the start of Tam administration (Fig. 6). *Defb3* and *Defb14* encode β-defensins, which are antimicrobial peptides primarily expressed in epithelial cells (46). The eyelids used for RNA sequencing in this study included not only the meibomian glands but also skin, subcutaneous tissue, orbicularis muscle, tarsal plate, and conjunctiva. The observed increases in *Defb3* and *Defb14* expression in *Elovl1* cKO mice likely originated from the conjunctival epithelium, possibly as a mechanism to enhance antimicrobial activity in the tear film in response to dry eye. *S100a8* and *S100a9*, which encode members of the S100 family of calcium-binding proteins, are expressed in neutrophils and monocytes and can be induced in epithelial cells under inflammatory conditions (47). *KRT16* (keratin 16) is known to be upregulated under inflammatory conditions and tissue damage (48). The late cornified envelope family proteins, LCE3s, are structural components of the stratum corneum and are involved in skin barrier formation (49). The small proline-rich proteins, SPRRs, are components of the cornified envelope (50), a protein cross-linked structure located near the surface of corneocytes. These results suggest that transcriptional changes in *Elovl1* cKO eyelids were mostly restricted to the conjunctival epithelium and the epidermal stratum corneum, whereas gene expression in meibocytes remained largely unchanged on day 30 after the start of Tam treatment.

## Discussion

In this study, we used *Tg-Elovl1* KO mice and *Elovl1* cKO mice to investigate the short-, medium-, and long-term effects of *Elovl1* disruption on meibum lipid composition and dry eye phenotypes. Previously, we revealed that the FA moiety in Chol-Es and the FAl moiety in WmEs were shortened in *Tg-Elovl1* KO mice (13, 38). Here, we found in *Tg-Elovl1* KO mice that, in addition to these, the FAl moiety in type 1ω WdiEs was shortened (Fig. 2) and further that type 2ω WdiEs, especially those containing monounsaturated diol–FAs, were reduced (Fig. 3, *B* and *C*). Furthermore, we revealed the time-dependent development of dry eye phenotypes (Fig. 5) and changes in the meibum lipid composition (Figure 6, Figure 7, Figure 8, Figure 9) following *Elovl1* disruption in *Elovl1* cKO mice.

In *Elovl1* cKO mice, most of the dry eye phenotypes were observable from day 10 after the start of Tam administration and had become more pronounced by day 30 (Fig. 5). The exception was corneal damage, which was not observed even on day 30. It is to be expected that it would take longer for abnormalities in the tear film lipid layer to cause corneal damage. In fact, corneal damage was not observed in *Tg-Elovl1* KO mice even at 9 weeks of age, but it was observed at 13 weeks of age (21). The most abundant lipid classes in meibum lipids are Chol-Es and WmEs, which together account for 60% to 92% of total meibum lipids, although this value varies between reports (16, 51, 52). Therefore, these compositional changes are likely to be the main contributors to the dry eye phenotypes observed in *Tg-Elovl1* KO and *Elovl1* cKO mice. Indeed, KO mice for *Soat1* and *Awat2*, which are involved in the production of Chol-Es and WmEs, respectively, exhibit severe dry eye phenotypes (37, 41). In contrast, the dry eye phenotypes in KO mice of *Cyp4f39*, which is involved in the production of ω-OH FA-containing lipid classes (type 1ω WdiEs, type 2ω WdiEs, OAHFAs, and Chol-OAHFAs), are milder (40).

By day 10 after the initiation of Tam administration, the timepoint at which the dry eye phenotypes became evident, the shortening of Chol-Es and WmEs had progressed sufficiently (Figs. 6 and 7). These chain shortenings were particularly prominent in saturated FA or FAl moieties relative to monounsaturated FA or FAl moieties. The percentage of ≥C24 saturated FA moieties in Chol-Es was 47% on day 0 and 11% on day 10, whereas that of ≥C24 saturated FAl moieties in WmEs was 96% on day 0 and 58% on day 10. This shortening proceeded in both cases through day 30 (Chol-Es, 4%; WmEs, 31%). It is highly likely that these changes caused the lower quality of the tear film lipid layer, leading to the dry eye phenotypes.

The fact that saturated FA/FAl moieties were more subject to shortening than monounsaturated FA/FAl moieties in Chol-Es and WmEs may be due to ELOVL1 contributing more to the elongation of saturated acyl-CoAs than to that of monounsaturated acyl-CoAs. ELOVL3, ELOVL4, and ELOVL7 are involved in FA elongation from C22 to C24 and from C24 to C26, and our previous *in vitro FA* elongation assay showed that their levels of activity relative to ELOVL1 were as follows: toward C22:0-CoA, ELOVL3 (17%); toward C22:1-CoA, ELOVL3 (27%); toward C24:0-CoA, ELOVL4 (26%); toward C24:1-CoA, ELOVL4 (60%), ELOVL3 (21%), and ELOVL7 (14%) (13, 18). Thus, ELOVL3, ELOVL4, and ELOVL7 show substantial activity toward monounsaturated acyl-CoAs, which may explain the relatively small effect of *Elovl1* KO on the production of monounsaturated C24:1 and C26:1 acyl-CoAs.

Similarly, differences in the effect of *Elovl1* KO according to the degree of unsaturation were also observed in the composition of type 2ω WdiEs (Fig. 3, *B* and *C*). In *Tg-Elovl1* KO mice, the quantity of monounsaturated diol–FA-containing type 2ω WdiEs was reduced to 43% of that in control mice, whereas there was either no or a smaller reduction in diunsaturated or triunsaturated diol–FA-containing type 2ω WdiEs (Fig. 3*C*). Most of the diol–FAs have C16:1 FA as their FA moiety (37, 40), indicating that the diol moieties of mono-, di-, and triunsaturated diol–FAs were saturated, monounsaturated, and diunsaturated, respectively. Since the chain lengths of diol–FA moieties were mainly C46–C52, the diol portions are calculated to be C30–C36. We suspect that *Elovl1* deficiency affected the elongation of saturated acyl-CoAs to C26:0, which caused reductions in the substrates for ELOVL4, leading to reduced production of the ELOVL4 products C30:0–C36:0-CoAs and their metabolites, saturated C30–C36 diols, and monounsaturated diol–FA-containing type 2ω WdiEs. However, the elongation of monounsaturated or diunsaturated acyl-CoAs to C26 may be less affected by the *Elovl1* KO, as described above.

**Figure 11:**
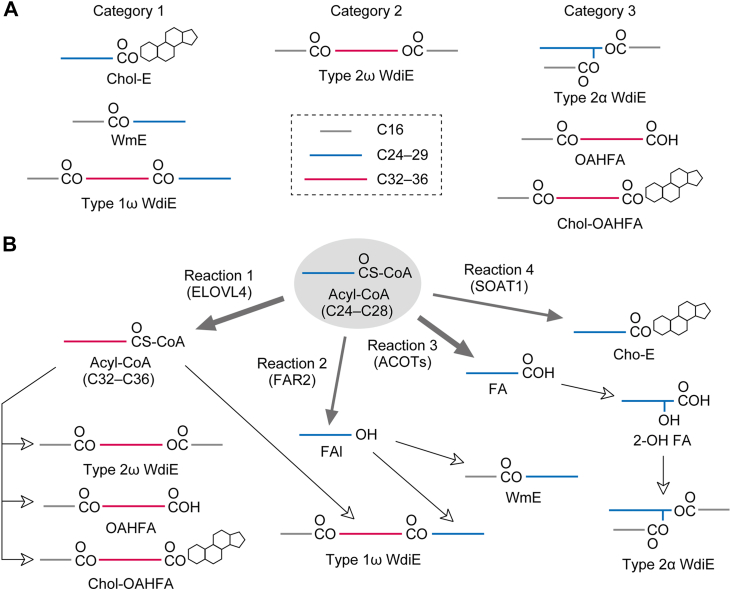
**Class-specific changes in meibum lipid composition caused by *Elovl1* KO**. *A*, classification of meibum lipids into three categories based on their sensitivity to *Elovl1* KO. Category 1 lipids (Chol-Es, WmEs, and type 1ω WdiEs) were substantially affected (shortened FA or FAl moiety) by *Elovl1* KO. Category 2 lipids (type 2ω WdiEs) were weakly affected (slightly reduced in quantity) by *Elovl1* KO. Category 3 lipids (type 2α WdiEs, OAHFAs, and Chol-OAHFAs) were little affected by *Elovl1* KO. Simplified structures of meibum lipids color coded by chain length are shown. *B*, metabolic pathway of C24–C28 acyl-CoAs, the products of ELOVL1, *via* four different reactions. The C24–C28 acyl-CoAs produced by ELOVL1 undergo one of the following four reactions. Reaction 1. Further elongation by ELOVL4. The products, C32–C36 acyl-CoAs, are metabolized to type 1ω WdiEs, type 2ω WdiEs, OAHFAs, or Chol-OAHFAs. Reaction 2. Reduction by FAR2. The products, C24–C28 FAls, are metabolized to type 1ω WdiEs and WmEs. Reaction 3. CoA hydrolysis by ACOTs. The products, C24–C28 FAs, are 2-hydroxylated and then metabolized to type 2α WdiEs. Reaction 4. Condensation with cholesterol by SOAT1. Of these four reactions, reactions 1 and 3 have high priority (*thick arrows*), whereas reactions 2 and 4 have low priority (*thin arrows*). Consequently, downstream metabolites of reactions 1 and 3 show low sensitivity to *Elovl1* KO, whereas those of reactions 2 and 4 show high sensitivity. ACOT, acyl-CoA thioesterase; Chol-E, cholesteryl ester; Chol-OAHFA, cholesteryl (*O*-acyl)-ω-hydroxy fatty acid; FA, fatty acid; FAl, fatty alcohol; OAHFA, (*O*-acyl)-ω-hydroxy fatty acid; 2-OH, 2-hydroxy; WdiE, wax diester; WmE, wax monoester.

We classified the meibum lipids into three categories based on the extent of the effects of *Elovl1* KO: category 1, those greatly affected (Chol-Es, WmEs, and type 1ω WdiEs); category 2, those slightly affected (type 2ω WdiEs); and category 3, those barely affected (type 2α WdiEs, OAHFAs, and Chol-OAHFAs) (Fig. 11*A*). In category 1 lipid classes, the chain lengths of the affected constituent FAs or FAls range from C24 to C28 (especially in saturated moieties), that is, they are derived from C24 to C28 acyl-CoAs, which are the direct products of ELOVL1. In contrast, the FA or FAl constituents in categories 2 and 3 have chain lengths of C30–C36, which are derived from C30–C36 acyl-CoAs, the products of ELOVL4. The exceptions to this were type 2α WdiEs, which were not affected by *Elovl1* KO despite containing C24–C28 diols (corresponding to C40–C44 diol–FAs) (Fig. 3*A*). C24–C28 acyl-CoAs, the products of ELOVL1, are used as substrates for four reactions: 1, FA elongation by ELOVL4; 2, reduction to FAls by FAR2; 3, hydrolysis of CoA (conversion to FAs) by acyl-CoA thioesterases (ACOTs); and 4, condensation with cholesterol by SOAT1. These reactions lead to the synthesis of distinct lipid classes: reaction 1, type 2ω WdiEs, OAHFAs, Chol-OAHFAs, and the diol moiety of type 1ω WdiEs; reaction 2, WmEs and the FAl moiety of type 1ω WdiEs; reaction 3, type 2α WdiEs; and reaction 4, Chol-Es (Fig. 11*B*). Reactions 2 and 4 are required for the production of category 1 lipid classes, whereas reactions 1 and 3 are needed for that of category 2 and 3 lipid classes. The different sensitivities of lipid classes to *Elovl1* KO (*i*.*e*., the aforementioned categorization) appear to be determined by the priority of the four reactions that the ELOVL1 products (C24–C28 acyl-CoAs) undergo: reactions 1 and 3 show a higher priority, whereas reactions 2 and 4 show a lower priority. FA elongation by ELOVL1 occurs in the ER (18). The reactions by ELOVL4 (reaction 1) and SOAT1 (reaction 4) also take place in the ER (18, 53). Reaction 3 is also probably catalyzed by ACOTs in the ER (ER–cytosol interface). Mammals have 13 ACOT isozymes (ACOT1–13) (54). Although it is unclear which ACOT catalyzes CoA hydrolysis from C24 to C28 acyl-CoAs to produce type 2α WdiEs, ACOT1, -7, -11, -12, and -13, which are localized in the cytosol, are likely candidates (54). Since the CoA moieties of C24–C28 acyl-CoAs produced in the ER are exposed to the cytosol, these ACOTs can attack them from the cytosol. In contrast, reaction 2, catalyzed by FAR2, is carried out in peroxisomes (55). Therefore, C24–C28 acyl-CoAs produced in the ER must be transported to peroxisomes for this reaction. The susceptibility of C24–C28 acyl-CoAs to these four reactions (*i*.*e*., the priority of the reactions) may be affected by the differences in the subcellular compartments in which the reactions occur. Furthermore, enzyme parameters such as *K*_m_, *V*_max_, and the specificity constant of each enzyme responsible for its respective reaction probably also affect the priority. We speculate that *Elovl1* KO (reduced C24–C28 acyl-CoA levels) has little effect on pathways with dominant metabolic flux (*i*.*e*., downstream of high-priority reactions such as reactions 1 and 3) but has a significant impact on pathways with less dominant metabolic flux (reactions 2 and 4).

Meibum lipids are characterized by longer carbon chain lengths than other lipids, a feature that is probably important for enhancing the hydrophobicity of the tear film lipid layer and thereby reducing water evaporation. The lipid layer also plays a role in providing appropriate viscoelasticity to the tear film (29). Lipids can exist in different phases—crystalline, gel, or liquid crystalline—depending on their composition and temperature. At the corneal surface temperature (32 °C), the presence of a liquid crystalline phase in meibum lipids has been reported (56). To maintain this liquid crystalline phase, the meibum lipids need to have an appropriate composition. The shortening of lipid chains in the meibum lipids caused by *Elovl1* KO may lead to reductions in the hydrophobicity and phase-transition temperature of the tear film lipid layer.

In this study, we used two types of *Elovl1* gene–engineered mice and revealed the relationship between changes in meibum lipid composition—particularly in terms of carbon chain length—and the dry eye phenotypes. We further demonstrated that the loss of *Elovl1* differentially influenced the composition of meibum lipids in a lipid class–dependent manner, thereby contributing to a deeper understanding of the molecular mechanisms underlying the complex metabolic pathways of meibum lipids. Despite recent advances, many aspects of meibum lipid metabolism remain poorly understood. In particular, the acyltransferases responsible for the production of type 2α WdiEs and Chol-OAHFAs have yet to be identified. Future investigations, including the identification of these enzymes, are essential for fully elucidating the metabolic pathways of meibum lipid production.

## Experimental procedures

### Mice

Details of the *Tg-Elovl1* KO mice and Tam-induced *Elovl1* cKO mice were described previously (13, 39). Deletion of *Elovl1* in *Elovl1* cKO mice was induced by intraperitoneally administering Tam (2 mg/day; Toronto Research Chemicals) dissolved in corn oil once daily for 5 consecutive days. Mice were housed in a specific pathogen-free animal-experiment facility under conditions of constant room temperature (23 ± 1 °C), humidity (50 ± 10%), and a 12 h light–dark cycle. Mice were given free access to water and a normal diet (CLEA Rodent Diet CE-2, CLEA Japan; PicoLab Rodent Diet 20, LabDiet). Given that previous studies have demonstrated no sex-related differences in the lipid composition of mouse meibum (57), we used a mixed population of male and female mice in this study. All animal experiments were approved by the Institutional Animal Care and Use Committee of Hokkaido University (permission no. 22-0036).

### Genomic PCR

The upper and lower eyelids of mice were prepared under a stereomicroscope (Stemi DV4, Carl Zeiss) and incubated overnight at 55 °C in 400 μl of lysis buffer (100 mM Tris–HCl [pH 8.5], 5 mM EDTA, 0.2% SDS, 200 mM NaCl, and 200 μg/ml proteinase K [Fujifilm Wako Pure Chemical Industries]). The samples were then centrifuged (9100*g*, room temperature, 5 min), and the supernatant was collected and mixed with 250 μl of 100% ethanol. After centrifugation (20,400*g*, room temperature, 5 min), the pellet was washed with 750 μl of 70% ethanol, centrifuged again, dried, and dissolved in 50 μl of TE (10 mM Tris–HCl [pH 8.0], 0.1 mM EDTA). The genomic DNA obtained was subjected to genomic PCR for genotyping as previously described (13, 39).

### Analyses of dry eye phenotypes

The frequency of eye blinks was counted as previously reported (37). Measurements of water evaporation from the ocular surface, BUT, and corneal damage scores were performed under anesthesia induced *via* intraperitoneal injection of pentobarbital (0.05 mg/g body weight; Tokyo Chemical Industry), as previously described (37).

### Lipid analyses *via* LC–MS/MS

Lipid extraction from mouse eyelids was carried out as follows. Upper and lower eyelids were excised from euthanized mice under a stereomicroscope and transferred into tubes containing zirconia beads (Tomy Seiko). The samples were then mixed with 600 μl of chloroform–methanol (1:2, v/v) and deuterium (*d*)-labeled lipid internal standards (5 nmol cholesteryl palmitate [C16:0 Chol-E-*d*_7_; Avanti Research] and 2 nmol lauryl oleate [C18:1 FA/C12:0 FAl WmE; Nu-Chek Prep]) and homogenized using a Micro Smash MS-100 (Tomy Seiko) at 4500 rpm and 4 °C for 1 min. After centrifugation (20,400*g*, 4 °C, 3 min), the supernatant was collected. The remaining pellets were subjected to re-extraction of lipids by adding 600 μl of chloroform–methanol (1:2, v/v) and homogenizing and centrifuging them. The two supernatants were combined and mixed with 400 μl of chloroform and 720 μl of water for phase separation. After centrifugation (20,400*g*, room temperature, 3 min), the organic phase was collected and dried. For measurements of WmEs and Chol-Es, samples were subjected to purification *via* TLC and hexane–methanol phase separation as previously described (37), except that TLC Silica gel 60 (Merck) was used as the TLC plate. Chol-OAHFAs and WdiEs were purified *via* hexane–water phase separation as follows. The dried samples were dissolved in 200 μl of hexane, mixed with 200 μl of water, and centrifuged (20,400*g*, room temperature, 3 min). The organic phase was collected and dried.

LC–MS/MS was performed using an LC-coupled tandem triple Q mass spectrometer (Xevo TQ-XS; Waters). Separation of lipids *via* LC was performed using a reversed-phase column (AQUITY UPLC CSH C18 column: particle size 1.7 μm, inner diameter 2.1 mm, length 100 mm; Waters) under the same conditions as previously described (37). Lipids were ionized *via* electrospray ionization, and positive ions were detected. MS/MS analyses were performed in MRM mode. The cone voltages were set as follows: Chol-Es, 15 V; WmEs, 40 V; type 2α/2ω WdiEs, 35 V; type 1ω WdiEs, 45 V; and Chol-OAHFAs, 35 V. The collision energies were set as follows: Chol-Es, 15 eV; WmEs, 15 eV (WmE containing C12:0 FAl) or 20 eV (others); type 2α/2ω WdiEs, 20 eV; type 1ω WdiEs, 20 eV; and Chol-OAHFAs, 15 eV (Chol-OAHFAs containing C32–C50 OAHFAs) or 20 eV (Chol-OAHFAs containing C51–C58 OAHFAs). The *m/z* values of precursor ions and product ions for Chol-Es, type 1ω WdiEs, and Chol-OAHFAs are shown in Tables S1–S3, and those of WmEs and type 2α/2ω WdiEs were as previously described (37). The quantity of each Chol-E and WmE species was calculated from the ratio to the peak area of the corresponding internal standard, after subtracting the peak area of the blank from that of the sample. Data analysis was performed using MassLynx software (Waters).

### Quantitative real-time RT–PCR

Eyelids were excised from mice, suspended in RNAlater (Merck), cut into small pieces using dissection scissors, and transferred to a tube containing zirconia beads. After the addition of 1 ml of TRIzol Reagent (Thermo Fisher Scientific), the eyelids were crushed using a Micro Smash MS-100 (5000 rpm, 4 °C, 1 min; three times at intervals of 1 min). Samples were then mixed with 200 μl of chloroform and centrifuged (12,000*g*, 4 °C, 15 min). The aqueous phase (upper layer) was collected, mixed with 500 μl of isopropanol, and incubated at room temperature for 10 min. RNAs were precipitated *via* centrifugation (12,000*g*, 4 °C, 10 min), washed with 1 ml of 75% ethanol, dried, and suspended in RNase-free water. The total RNAs thus obtained were converted to complementary DNAs using the PrimeScript II 1st strand cDNA Synthesis Kit (Takara Bio) and an oligo dT primer (Takara Bio), according to the manufacturer’s instructions, and subjected to quantitative real-time RT–PCR using KOD SYBR qPCR Mix (Toyobo) and a primer pair specific to each gene on a CFX96 Touch Real-Time PCR Detection System (Bio-Rad). The primers used were as previously described (37, 39, 58), except for the primer set for *Scd1* (forward primer, 5′-CCTCCGGAAATGAACGAGAGAA-3′; reverse primer, 5′-TCCTCCAGACGTACTCCAGC-3′), which was created in this study. The mRNA levels were normalized with respect to *Hprt1* (hypoxanthine phosphoribosyltransferase 1).

### RNA sequencing

Total RNAs prepared from eyelid tissue using TRIzol Reagent as described above were purified with the NucleoSpin RNA II Kit (Takara Bio), according to the manufacturer’s protocol. RNA sequencing was performed as previously described (39), except that the sequencing platform was changed from Illumina NovaSeq 6000 (Illumina) to Illumina NovaSeq X Plus (Illumina), and the version of FastQC (http://www.bioinformatics.babraham.ac.uk/projects/fastqc/) was updated from 0.11.7 to 0.12.1.

### Statistical analysis

Data are presented as mean + SD or mean ± SD. Welch’s unpaired two-tailed *t* test was performed using Microsoft Excel (Microsoft) or Prism (Dotmatics). A *p* value of less than 0.05 was considered statistically significant.

## Data availability

All data generated or analyzed during this study are contained within the article.

## Supporting information

This article contains supporting information.

## Conflict of interest

The authors declare that they have no conflicts of interest with the contents of this article.
