# Supplementary material for: Time-dependent changes in meibum lipid composition and progression of dry eye following disruption of the fatty acid elongase Elovl1
Source: J Biol Chem. 2026 Jan 13;302(3):111160. doi: 10.1016/j.jbc.2026.111160 (PMC12887387; doi:10.1016/j.jbc.2026.111160)
Supplement: Supplementary Material [file mmc1.pdf]

Time-dependent changes in meibum lipid composition and progression of dry eye following disruption of the fatty acid elongase *Elovl1*

Himari Tada, Keisuke Jojima, Taiga Hiranuma, Takayuki Sassa, and Akio Kihara

Table S1. Chol-E MRM parameters (Q1 and Q3).

FA moiety	Precursor ion (Q1) $[M + NH_4]^+$	Product ion (Q3) $[Cholesterol - H_2O]^+$
d_7-C16:0	649.5	376.4
C18:0	670.5	369.4
C19:0	684.5	369.4
C20:0	698.6	369.4
C21:0	712.6	369.4
C22:0	726.6	369.4
C23:0	740.6	369.4
C24:0	754.6	369.4
C25:0	768.6	369.4
C26:0	782.6	369.4
C27:0	796.7	369.4
C28:0	810.7	369.4
C16:1	640.5	369.4
C17:1	654.5	369.4
C18:1	668.5	369.4
C19:1	682.5	369.4
C20:1	696.5	369.4
C21:1	710.6	369.4
C22:1	724.6	369.4
C23:1	738.6	369.4
C24:1	752.6	369.4
C25:1	766.6	369.4
C26:1	780.6	369.4
C27:1	794.6	369.4
C28:1	808.7	369.4
C29:1	822.7	369.4
C30:1	836.7	369.4
C31:1	850.7	369.4

C32:1	864.7	369.4
C33:1	878.7	369.4
C34:1	892.7	369.4
C35:1	906.8	369.4
C36:1	920.8	369.4

Table S2. Type 1ω WdiE MRM parameters (Q1 and Q3).

OAHFA moiety	FAl moiety	Precursor ion (Q1) $[M + H]^+$	Product ion (Q3) $[M + H - FAl]^+$
C50:3	C16:0	981.9	739.6
C50:3	C17:0	996.0	739.6
C50:3	C18:0	1010.0	739.6
C50:3	C19:0	1024.0	739.6
C50:3	C20:0	1038.0	739.6
C50:3	C21:0	1052.0	739.6
C50:3	C22:0	1066.0	739.6
C50:3	C23:0	1080.0	739.6
C50:3	C24:0	1094.1	739.6
C50:3	C25:0	1108.1	739.6
C50:3	C27:0	1136.1	739.6
C50:3	C28:0	1150.1	739.6
C50:3	C29:0	1164.1	739.6
C50:3	C30:0	1178.1	739.6
C50:3	C31:0	1192.2	739.6
C50:3	C32:0	1206.2	739.6
C50:3	C33:0	1220.2	739.6
C50:3	C34:0	1234.2	739.6
C50:3	C35:0	1248.2	739.6
C50:3	C36:0	1262.2	739.6
C50:3	C16:1	979.9	739.6
C50:3	C17:1	993.9	739.6
C50:3	C18:1	1008.0	739.6
C50:3	C19:1	1022.0	739.6
C50:3	C20:1	1036.0	739.6
C50:3	C21:1	1050.0	739.6
C50:3	C22:1	1064.0	739.6
C50:3	C23:1	1078.0	739.6

C50:3	C24:1	1092.0	739.6
C50:3	C25:1	1106.1	739.6
C50:3	C26:1	1120.1	739.6
C50:3	C27:1	1134.1	739.6
C50:3	C28:1	1148.1	739.6
C50:3	C29:1	1162.1	739.6
C50:3	C30:1	1176.1	739.6
C50:3	C31:1	1190.1	739.6
C50:3	C32:1	1204.2	739.6
C50:3	C33:1	1218.2	739.6
C50:3	C34:1	1232.2	739.6
C50:3	C35:1	1246.2	739.6
C50:3	C36:1	1260.2	739.6
C32:1	C26:0	873.9	491.4
C33:1	C26:0	887.9	505.4
C34:1	C26:0	901.9	519.4
C35:1	C26:0	915.9	533.4
C36:1	C26:0	929.9	547.4
C37:1	C26:0	943.9	561.4
C38:1	C26:0	957.9	575.4
C39:1	C26:0	972	589.5
C40:1	C26:0	986	603.5
C41:1	C26:0	1000	617.5
C42:1	C26:0	1014	631.5
C43:1	C26:0	1028	645.5
C44:1	C26:0	1042	659.5
C45:1	C26:0	1056	673.5
C46:1	C26:0	1070.1	687.6
C47:1	C26:0	1084.1	701.6
C48:1	C26:0	1098.1	715.6
C49:1	C26:0	1112.1	729.6
C50:1	C26:0	1126.1	743.6

C51:1	C26:0	1140.1	757.6
C52:1	C26:0	1154.1	771.6
C53:1	C26:0	1168.2	785.7
C54:1	C26:0	1182.2	799.7
C55:1	C26:0	1196.2	813.7
C56:1	C26:0	1210.2	827.7
C32:2	C26:0	871.8	489.3
C33:2	C26:0	885.8	503.4
C34:2	C26:0	899.8	517.4
C35:2	C26:0	913.8	531.4
C36:2	C26:0	927.9	545.4
C37:2	C26:0	941.9	559.4
C38:2	C26:0	955.9	573.4
C39:2	C26:0	969.9	587.4
C40:2	C26:0	983.9	601.5
C41:2	C26:0	997.9	615.5
C42:2	C26:0	1011.9	629.5
C43:2	C26:0	1026	643.5
C44:2	C26:0	1040	657.5
C45:2	C26:0	1054	671.5
C46:2	C26:0	1068	685.5
C47:2	C26:0	1082	699.6
C48:2	C26:0	1096	713.6
C49:2	C26:0	1110.1	727.6
C50:2	C26:0	1124.1	741.6
C51:2	C26:0	1138.1	755.6
C52:2	C26:0	1152.1	769.6
C53:2	C26:0	1166.1	783.6
C54:2	C26:0	1180.1	797.7
C55:2	C26:0	1194.2	811.7
C56:2	C26:0	1208.2	825.7
C32:3	C26:0	869.8	487.3

C33:3	C26:0	883.8	501.3
C34:3	C26:0	897.9	515.4
C35:3	C26:0	911.9	529.4
C36:3	C26:0	925.9	543.4
C37:3	C26:0	939.9	557.4
C38:3	C26:0	953.9	571.4
C39:3	C26:0	967.9	585.4
C40:3	C26:0	981.9	599.4
C41:3	C26:0	996	613.5
C42:3	C26:0	1010	627.5
C43:3	C26:0	1024	641.5
C44:3	C26:0	1038	655.5
C45:3	C26:0	1052	669.5
C46:3	C26:0	1066	683.5
C47:3	C26:0	1080	697.5
C48:3	C26:0	1094.1	711.6
C49:3	C26:0	1108.1	725.6
C50:3	C26:0	1122.1	739.6
C51:3	C26:0	1136.1	753.6
C52:3	C26:0	1150.1	767.6
C53:3	C26:0	1164.1	781.6
C54:3	C26:0	1178.1	795.6
C55:3	C26:1	1192.2	809.7
C56:3	C26:2	1206.2	823.7

Table S3. Chol-OAHFA MRM parameters (Q1 and Q3).

OAHFA moiety	Precursor ion (Q1) $[M + H]^+$	Product ion (Q3) [Cholesterol − $H_2O]^+$
C46:1	1074.1	369.4
C47:1	1088.1	369.4
C48:1	1102.1	369.4
C49:1	1116.1	369.4
C50:1	1130.1	369.4
C51:1	1144.2	369.4
C52:1	1158.2	369.4
C53:1	1172.2	369.4
C54:1	1186.2	369.4
C55:1	1200.2	369.4
C56:1	1214.2	369.4
C46:2	1072.1	369.4
C47:2	1086.1	369.4
C48:2	1100.1	369.4
C49:2	1114.1	369.4
C50:2	1128.1	369.4
C51:2	1142.1	369.4
C52:2	1156.2	369.4
C53:2	1170.2	369.4
C54:2	1184.2	369.4
C55:2	1198.2	369.4
C56:2	1212.2	369.4
C46:3	1070.1	369.4
C47:3	1084.1	369.4
C48:3	1098.1	369.4
C49:3	1112.1	369.4
C50:3	1126.1	369.4
C51:3	1140.1	369.4

C52:3	1154.1	369.4
C53:3	1168.2	369.4
C54:3	1182.2	369.4
C55:3	1196.3	369.4
C56:3	1210.3	369.4
